# *α*-TEA cooperates with MEK or mTOR inhibitors to induce apoptosis via targeting IRS/PI3K pathways

**DOI:** 10.1038/sj.bjc.6606019

**Published:** 2010-11-30

**Authors:** R Tiwary, W Yu, B G Sanders, K Kline

**Affiliations:** 1 School of Biological Sciences/C0900, University of Texas, 1 University Station, Austin, TX 78712, USA;; 2 Department of Nutritional Sciences/A2703, University of Texas, 1 University Station, Austin, TX 78712, USA

**Keywords:** *α*-TEA, Akt, breast cancer, ERK, IRS-1, mTOR

## Abstract

**Background:**

*α*-Tocopherol ether-linked acetic acid (*α*-TEA) is a promising agent for cancer prevention/therapy based on its antitumour actions in a variety of cancers.

**Methods:**

Human breast cancer cells, MCF-7 and HCC-1954, were used to study the effect of *α*-TEA using Annexin V/PI staining, western blot analyses, and siRNA knockdown techniques.

**Results:**

*α*-Tocopherol ether-linked acetic acid suppressed constitutively active basal levels of pAKT, pERK, pmTOR, and their downstream targets, as well as induced both cell types to undergo apoptosis. Phosphoinositide 3-kinase (PI3K) inhibitor wortmannin suppressed pAKT, pERK, pmTOR, and their downstream targets, indicating PI3K to be a common upstream mediator. In addition, *α*-TEA induced increased levels of pIRS-1 (Ser-307), a phosphorylation site correlated with insulin receptor substrate-1 (IRS-1) inactivation, and decreased levels of total IRS-1. Small interfering RNA (siRNA) knockdown of JNK blocked the impact of *α*-TEA on pIRS-1 and total IRS-1 and impeded its ability to downregulate the phosphorylated status of AKT, ERK, and mTOR. Combinations of *α*-TEA+MEK or mTOR inhibitor acted cooperatively to induce apoptosis and reduce basal levels of pERK and pmTOR. Importantly, inhibition of MEK and mTOR resulted in increased levels of pAKT and IRS-1, and *α*-TEA blocked them.

**Conclusions:**

Downregulation of IRS-1/PI3K pathways via JNK are critical for *α*-TEA and *α*-TEA+MEK or mTOR inhibitor-induced apoptosis in human MCF-7 and HCC-1954 breast cancer cells.

The PI3K, AKT, ERK, and mTOR prosurvival mediators are important therapeutic targets, as they are constitutively activated in many cancers and contribute to cancer progression by promoting cellular proliferation and inhibiting cell death signalling pathways (Falasca, 2010). Phosphoinositide 3-kinase (PI3K) is activated at the cell membrane by tyrosine kinase growth factor receptors, such as members of the epidermal growth factor receptor family (EGFR and Her-2), and by the insulin-like growth factor-1 receptor (IGFR), as well as its downstream signalling substrate IRS-1 (insulin receptor substrate-1; Schlessinger, 2000). Phosphoinositide 3-kinase promotes cancer cell survival by activation of downstream mediators AKT and Ras, the latter leading to ERK activation (McCubrey *et al*, 2007). AKT exerts its survival role via a diverse array of substrates, which control key cellular processes, including apoptosis, cell cycle progression, transcription, and translation (Chang *et al*, 2003). A major downstream substrate of AKT is the serine/threonine kinase mTOR. AKT can directly phosphorylate mTOR at ser-2448 and activate it, as well as cause indirect activation of mTOR by phosphorylating and inactivating TSC2 (tuberous sclerosis complex 2, also called tuberin). The raptor–mTOR complex signals to its downstream effectors S6 kinase/ribosomal protein S6 (p70S6K) and the eIF4E-binding protein (p4E-BP1) to control transcription and translation, which selectively regulates multiple proteins that control cell cycle and apoptosis (Gibbons *et al*, 2009). Additionally, AKT can directly regulate apoptosis by phosphorylating and inactivating proapoptotic proteins such as Bad and caspase-9 (Datta *et al*, 1997; Cardone *et al*, 1998; Mabuchi *et al*, 2002). Extracellular signal-regulated kinase (ERK) exerts its antiapoptotic effects by phosphorylating and inactivating Bad (Mabuchi *et al*, 2002). As with most intracellular signalling cascades, cross-talk and negative and positive feedback loops complicate final signalling outcomes.

For example, the mTOR substrate p70S6K can ultimately diminish prosurvival signalling via PI3K/AKT by catalysing an inhibitory phosphorylation site on insulin receptor substrate-1, an upstream mediator of PI3K (Wan *et al*, 2007). Likewise, ERK can diminish prosurvival signalling by PI3K/AKT via p70S6K (Jiang *et al*, 2009). Therefore, although ERK and mTOR showed potential as anticancer targets, inhibitors of ERK or mTOR alone are limited in clinical application because of the mitigation of these negative feedback loops essential for controlling AKT activity (Sun *et al*, 2005). Thus, this more in-depth understanding of signalling pathways suggests that ERK or mTOR inhibitors need to be combined with agents that can circumvent the loss of negative feedback controls on AKT and/or effectively block AKT activity.

*α*-Tocopherol ether-linked acetic acid (*α*-TEA) is a promising agent for cancer prevention/therapy based on its antitumour actions reported in several *in vitro* and *in vivo* studies on a variety of cancers, including human oestrogen-responsive and nonresponsive breast cancers (Anderson *et al*, 2002; Lawson *et al*, 2004; Shun *et al*, 2004; Hahn *et al*, 2006; Yu *et al*, 2006; Jia *et al*, 2008a, 2008b; Wang *et al*, 2008; Hahn *et al*, 2009; Shun *et al*, 2010). These previous studies showed that *α*-TEA induces apoptosis in human breast cancer cells via activation of proapoptotic extrinsic death receptor Fas and DR5 as well as activation of a JNK/p73/Noxa pathway, leading to activation of caspase-8 and mitochondrial-dependent apoptosis (Shun *et al*, 2004; Wang *et al*, 2008; Yu *et al*, 2010). In this study, we demonstrate that *α*-TEA induces apoptosis not only by activation of pro-apoptotic pathways but also by targeting IRS-1/PI3K growth/survival pathways to suppress multiple prosurvival factors via c-Jun N-terminal kinase (JNK). Lessons learned from this study are that *α*-TEA in combination with ERK or mTOR inhibitors is an effective anticancer strategy and lays the groundwork for a mechanism-based combinational therapeutic strategy for employing ERK and mTOR inhibitors more effectively in the clinic.

## Materials and methods

### Chemicals

*α*-Tocopherol ether-linked acetic acid (FW=488.8) was prepared in-house as described previously (Lawson *et al*, 2004). Map kinase kinase (MEK) inhibitor (U01260) and PI3K inhibitor (wortmannin) were purchased from Cell Signaling Technology (Beverly, MA, USA). Mammalian target of rapamycin (mTOR) inhibitor (rapamycin) and JNK inhibitor (SP600125) were purchased from Calbiochem (La Jolla, CA, USA).

### Cell culture

The oestrogen receptor-negative human breast cancer cells, HCC-1954, were obtained from the American Type Culture Collection (Manassas, VA, USA). The oestrogen-responsive human breast cancer cells, MCF-7, were originally provided by Dr Suzanne Fuqua (Baylor College of Medicine, Houston, TX, USA). The MCF-7 cells were cultured as previously described (Wang *et al*, 2008). The HCC-1954 cells were cultured in RPMI media with 10% FBS. For experiments, FBS was reduced to 2% and cells were allowed to attach overnight before treatments. *α*-Tocopherol ether-linked acetic acid (40 mM) dissolved in ethanol served as stock solution. Equivalent final concentrations of ethanol (0.05–0.1%) for concentration of *α*-TEA used served as vehicle controls (VEH).

### Western blot analyses

Whole-cell protein lysates and western blot analyses were conducted as described previously (Jia *et al*, 2008a, 2008b). Proteins at 20–50 *μ*g per lane were separated by SDS–PAGE and transferred to nitrocellulose (Optitran BA-S supported nitrocellulose; Schleicher and Schuell, Keene, NH, USA). Antibodies to the following proteins were used: poly (ADP-ribose) polymerase (PARP), phospho-JNK (pJNK thr-183/tyr-185), phospho-ERK (pERK thr-202/tyr-204), total ERK and phospho-caspase-9 (pcaspase-9 ser-196) (Santa Cruz Biotechnology, Santa Cruz, CA, USA), Phospho-AKT (pAKT ser-473), pAKT (ser-308), total AKT, phospho-GSK (pGSK *α*/*β* ser-21/9), total GSK *α*/*β*, phospho-Bad (pBad ser-136), phospho-Bad (pBad ser-112), total Bad, total casapse-9, phospho-p90RSK (pp90RSK ser-380), total p90RSK, phospho-mTOR (pmTOR ser-2448), total mTOR, phospho-p70S6K (pp70S6K thr-389), total p70S6K, phospho-4E-BP1 (p4E-BP1 thr-37/46), total 4E-BP1, phospho-IRS (pIRS ser-307), total IRS, and glyceraldehyde-3-phosphate dehydrogenase (GAPDH; Cell Signaling Technology).

### Small interfering RNA (siRNA) transfection

A scrambled RNA duplex that does not target any known genes was used as a nonspecific negative control for RNAi (referred to as control siRNA) (Ambion, Austin, TX, USA). Transfection of siRNAs to AKT1, AKT2, JNK1/2, or control (Ambion) was performed in 100 mm^2^ cell culture dishes at a density of 2 × 10^6^ cells per dish using Lipofectamine-2000 (Invitrogen, Carlsbad, CA, USA) and siRNA duplex, resulting in a final siRNA concentration of 30 nM following the company's instructions. At 1 day after transfection, the cells were re-cultured in 100 mm^2^ dishes at 2 × 10^6^ cells per dish and incubated for 1 day followed by treatments.

### Quantification of apoptosis

Apoptosis was quantified by Annexin V-FITC/propidium iodide (PI) assay following the manufacturer's instructions. The Annexin V-FITC/PI assay measures the amount of phosphatidylserine on the outer surface of the plasma membrane (a biochemical alteration unique to membranes of apoptotic cells) and the amount of PI, a dye that does not cross the plasma membrane of viable cells but readily enters dead cells or cells in the late stages of apoptosis and binds DNA. Fluorescence was measured using fluorescence activated cell sorter (FACS) analyses with a FACSCalibur flow cytometer, and data were analysed using CellQuest software (BD Biosciences, San Jose, CA, USA). Cells displaying phosphatidylserine on their surface (i.e., positive for annexin-V fluorescence) were considered to be apoptotic.

### Statistical analysis

Apoptosis data were analysed using a one-way analysis of variance (ANOVA) followed by Tukey's test for comparison of more than two treatments or a two-tailed Student's *t*-test for comparison between two treatments to determine statistical differences. Differences were considered statistically significant at *P*<0.05.

## Results

### *α*-TEA induces apoptosis in HCC-1954 and MCF-7 human breast cancer cells

**Figure 1:**
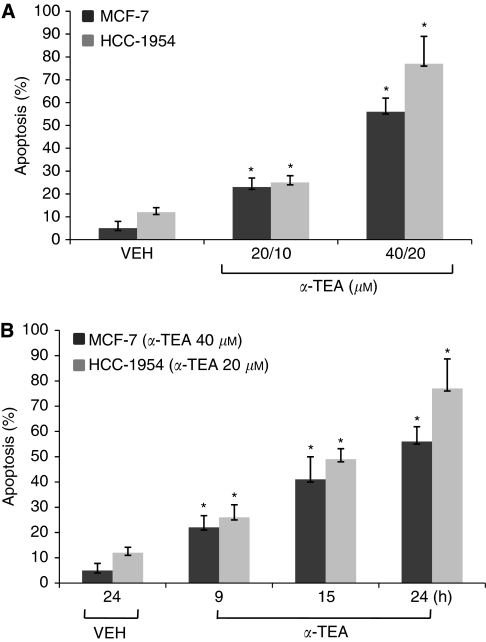
*α*-Tocopherol ether-linked acetic acid (*α*-TEA) induces apoptosis in human breast cancer cells in a dose- and time-dependent manner. (**A**) MCF-7 and HCC-1954 cells were treated with different concentrations of *α*-TEA for 24 h and the proapoptotic property of *α*-TEA was evaluated by FACS/Annexin V assay. (**B**) MCF-7 and HCC-1954 cells were treated with 40 and 20 *μ*M *α*-TEA, respectively, for different times, and the proapoptotic property of *α*-TEA was evaluated by FACS/Annexin V assay. Data are the mean±s.d. of different independent experiments. ^*^Significantly different from VEH (*P*<0.05).

Human breast cancer cells, MCF-7 and HCC-1954, were treated with *α*-TEA at different doses (Figure 1A) or for different periods of time (Figure 1B). Apoptosis was quantified by FACS analyses of cells labelled with FITC-Annexin V. Treatment of MCF-7 cells with *α*-TEA at 20 or 40 *μ*M and HCC-1954 cells at 10 or 20 *μ*M for 24 h induced both cell types to undergo apoptosis in a dose-dependent manner, with HCC-1954 cells exhibiting greater sensitivity than MCF-7 cells (Figure 1A). Treatment of the MCF-7 and HCC-1954 cells with *α*-TEA at 40 and 20 *μ*M, respectively, for 9, 15, and 24 h induced apoptosis in a time-dependent manner (Figure 1B). These data show that *α*-TEA is a potent apoptotic inducer of both ER-responsive and nonresponsive human breast cancer cells.

### *α*-TEA reduces high basal levels of phosphorylated AKT, ERK1/2, and mTOR

**Figure 2:**
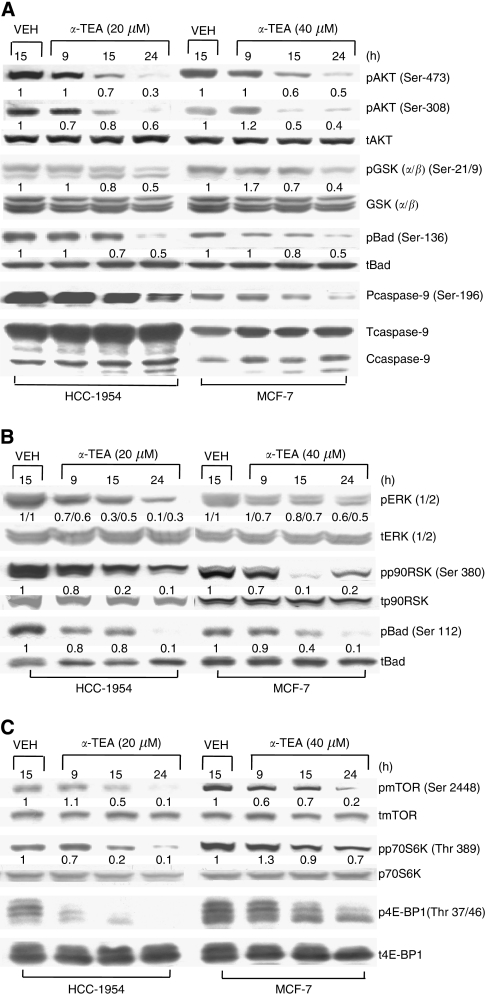
*α*-Tocopherol ether-linked acetic acid (*α*-TEA) suppresses AKT, ERK, mTOR, as well as their downstream targets. HCC-1954 and MCF-7 cells were treated with 20 and 40 *μ*M *α*-TEA, respectively, for 9, 15, and 24 h. (**A**) Protein levels of pAKT (Ser-473 and Ser-308), pGSK (*α*/*β*) (Ser 21/9), pBad (Ser-136), pcaspase-9 (Ser-196), as well as levels of total AKT, GSK, Bad, and caspase-9 were determined by western blot analyses. (**B**) Protein levels of pERK1/2, pp90RSK (Ser-380), and pBad (Ser-112), as well as total ERK, p90RSK, and Bad were determined by western blot analyses. (**C**) Protein levels of pmTOR (Ser-2448), pp70S6K (Thr-389), and p4E-BP1 (Thr-37/46), and levels of total mTOR, p70S6K, and 4E-BP1 were determined by western blot analyses. Data are representative of two separate experiments.

**Figure 3:**
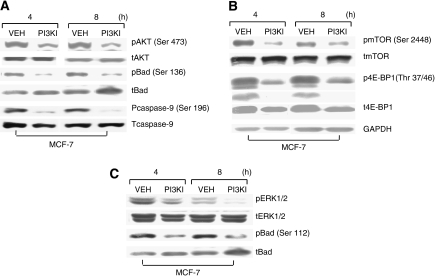
Extracellular signal-regulated kinase (ERK) and mTOR are downstream targets of PI3K. MCF-7 cells were treated with 1 *μ*M PI3K inhibitor wortmannin for 4 and 8 h. (**A**) Protein levels of pAKT (Ser-473), pBad (Ser-136), and pcaspase-9 (Ser-196), and levels of total AKT, Bad, and caspase-9 were determined by western blot analyses. (**B**) Protein levels of pERK1/2 and pBad (Ser-112) and total levels of ERK1/2 and Bad were determined by western blot analyses. (**C**) Protein levels of pmTOR (Ser-2448) and p4E-BP1 (Thr-37/46), and total levels of mTOR and 4E-BP1 were determined by western blot analyses. Data are representative of two separate experiments.

Vehicle-treated HCC-1954 and MCF-7 cells express high levels of active AKT (pAKT) and phosphorylated downstream substrates GSK (pGSK), Bad (pBad), and caspase 9 (pcaspase-9). Treatment of HCC-1954 (20 *μ*M) and MCF-7 cells (40 *μ*M) with *α*-TEA for 9, 15, and 24 h: (1) reduces levels of pAKT (both Ser-473 and Ser-308) as well as its downstream substrates pGSK (*α*/*β*) (Ser-21/9), pBad (Ser-136), and pcaspase-9 (Ser-196) and increases cleaved caspase 9 in a time-dependent manner (Figure 2A), (2) downregulates pERK1/2 and its substrates pp90SK (Ser-380) and pBad (Ser-112) (Figure 2B), and (3) downregulates pmTOR (Ser-2448) and its substrates pp70S6K (Thr-389) and p4E-BP1 (Thr-37/46) (Figure 2C). These data show that *α*-TEA suppresses the active forms of AKT, ERK, mTOR, and their downstream substrates, resulting in activation of proapoptotic mediators Bad and caspase-9.

### PI3K inhibitor (wortmannin) suppresses pAKT, pERK1/2, and pmTOR in MCF-7 cells

To better understand how the basal levels of pAKT, pERK, and pmTOR are regulated, MCF-7 cells were treated with 1 *μ*M wortmannin (PI3KI) for 4 and 8 h. Wortmannin reduced the phosphorylated forms of: (1) AKT and its substrates pBad (Ser-136) and pcaspase-9 (Ser-196) (Figure 3A), (2) ERK1/2 and its substrate pBad (Ser-112) (Figure 3B), and pmTOR (Ser-2448) and its substrate p4E-BP1 (Thr 37/46) (Figure 3C), indicating that PI3K is a major contributor to the basal levels of AKT, ERK, and mTOR.

### *α*-TEA induces increased levels of phospho-IRS-1 (Ser-307) and decreased levels of total IRS-1 via JNK

**Figure 4:**
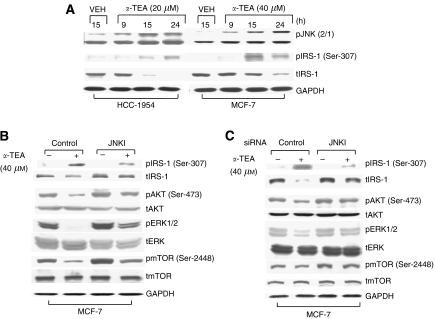
*α*-Tocopherol ether-linked acetic acid (*α*-TEA) downregulates AKT, ERK, and mTOR via JNK-mediated decrease in IRS protein expression. (**A**) HCC-1954 and MCF-7 cells were treated with 20 and 40 *μ*M *α*-TEA, respectively, for 9, 15, and 24 h. Western blot analyses were performed to detect phospho-JNK2/1, pIRS-1 (Ser-307), and total IRS, with GAPDH serving as loading control. (**B**) MCF-7 cells were treated with 10 *μ*M JNK inhibitor SP600125 (JNKI)+40 *μ*M *α*-TEA for 18 h. Protein levels of pAKT (Ser-473), pERK1/2, pmTOR (Ser-2448), pIRS-1 (Ser-307), and total levels of AKT, ERK1/2, mTOR, and IRS-1 were determined by western blot analyses, with GAPDH serving as lane control. (**C**) MCF-7 cells transfected with siRNA to JNK or control siRNA were treated with 40 *μ*M *α*-TEA for 18 h. Protein levels of pAKT (Ser-473), pERK1/2, pmTOR (Ser-2448), pIRS-1 (Ser-307), and levels of total AKT, ERK, mTOR, and IRS were determined by western blot analyses, with GAPDH as lane control. Data are representative of two separate experiments.

To identify a common upstream factor that might account for how *α*-TEA suppresses pAKT, pERK, and pmTOR in unison, the effect of *α*-TEA on phospho-IRS-1 (pIRS-1 Ser-307) was examined. Ser-307 is a phosphorylation site regulated by JNK for inactivation of IRS (Mamay *et al*, 2003) via reduction of total IRS protein levels (Hiratani *et al*, 2005). As *α*-TEA has been reported to induce a prolonged activation of JNK (Shun *et al*, 2004; Yu *et al*, 2006; Jia *et al*, 2008a, 2008b; Wang *et al*, 2008), IRS-1 may be a promising upstream target for *α*-TEA-mediated events. The HCC-1954 and MCF7 cells were treated with 20 and 40 *μ*M *α*-TEA, respectively, for 9, 15, and 24 h. *α*-Tocopherol ether-linked acetic acid induced increased levels of pJNK2/1, increased levels of pIRS-1 (ser-307), and reduced levels of total IRS-1 in both cell lines (Figure 4A). Knockdown of JNK using a chemical inhibitor SP600125 (JNKI) (Figure 4B) or siRNA (Figure 4C) blocked the ability of *α*-TEA to increase pIRS-1 (Ser-307) and decrease levels of total IRS-1 protein, and suppressed the ability of *α*-TEA to reduce pAKT (Ser-473), pERK1/2, and pmTOR (Ser-2448). These data suggest that downregulation of IRS-1 is involved in the ability of *α*-TEA to suppress AKT, ERK, and mTOR signalling and demonstrated that JNK is involved in this event via phosphorylation of IRS-1 at Ser-307, leading to its degradation.

### Inhibitors of PI3K, MEK/ERK, and mTOR as well as siRNA to AKT1 and AKT2 cooperated with *α*-TEA to induce apoptosis

**Figure 5:**
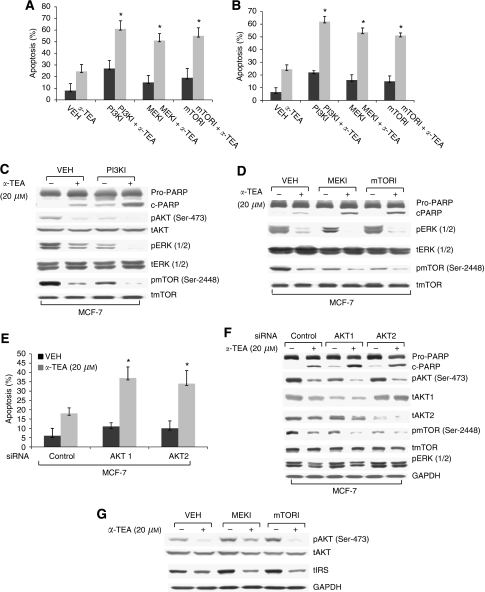
Inhibitors of PI3K, MEK/ERK, mTOR, as well as siRNAs to AKT1 and AKT2 enhance *α*-TEA-induced apoptosis. (**A** and **B**) HCC-1954 and MCF-7 cells were treated with 1 *μ*M PI3K inhibitor wortmannin (PI3KI), 10 *μ*M MEK inhibitor U01260 (MEKI), or 50 nM mTOR inhibitor rapamycin (mTORI) plus 10 and 20 *μ*M of *α*-TEA for 18 h, respectively. Apoptosis was determined by Annexin V/PI staining. (**C** and **D**) Protein levels of cleaved PARP, pAKT (Ser-473), pERK1/2, and pmTOR (Ser-2448) as well as levels of total AKT, ERK1/2, and mTOR were determined by western blot analyses in MCF-7 cells treated with the three inhibitors plus *α*-TEA. (**E**) MCF-7 cells transfected with siRNAs to AKT1, AKT2, or control was treated with 20 *μ*M *α*-TEA for 18 h. Apoptosis was determined by Annexin V/PI staining. (**F**) Protein levels of cleaved PARP, pAKT (Ser-473), pmTOR (Ser-2448), pERK1/2, and levels of total AKT1, AKT2, and mTOR were determined by western blot analyses. Levels of GAPDH served as lane controls. (**G**) MCF-7 cells treated with 10 *μ*M MEK inhibitor U01260 (MEKI) or 50 nM mTOR inhibitor rapamycin (mTORI) plus 20 *μ*M of *α*-TEA for 18 h. Protein levels of pAKT (Ser-473), AKT, IRS-1, and GAPDH were determined by western blot analyses. Data in (**C**, **D**, **F**, and **G**) are representative of two separate experiments. Data in (**A**, **B**, and **E**) are presented as mean±s.d. of three independent experiments. ^*^Significantly different from control (*P*<0.05).

**Figure 6:**
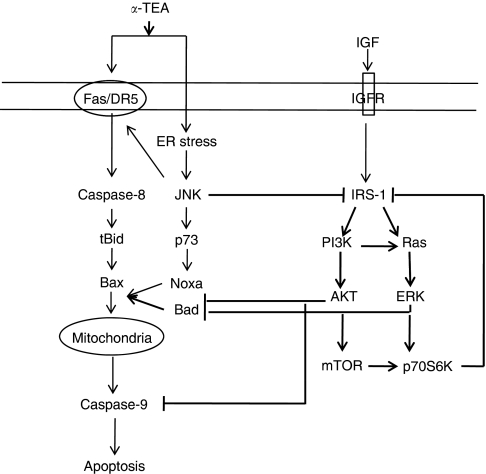
Schematic diagram depicting signalling pathways that are involved in *α*-TEA alone and *α*-TEA+MEK or mTOR inhibitor-induced apoptosis. Schematic diagram depicts cross-talk between survival and death signalling pathways in *α*-TEA alone and *α*-TEA+MEK or mTOR inhibitor-induced apoptosis in human breast cancer cells. Constitutively active insulin receptor substrate-1 (IRS-1) activates AKT and ERK via phosphorylations mediated by PI3K and Ras, respectively, leading to subsequent phosphorylation events that inhibit proapoptotic mediators Bad and caspase 9, and activate prosurvival mTOR. Beneficial antitumour actions by inhibitors of mTOR (rapamycin) and ERK (MEK inhibitor) are compromised by elimination of mTOR or ERK-regulated p70S6K negative loop inhibiting IRS-1 signalling. The loss of this negative feedback loop results in continued activation of AKT signalling and inhibition of apoptosis by inactivation of Bad and caspase 9. *α*-Tocopherol ether-linked acetic acid (*α*-TEA) induces death receptor-mediated apoptosis (depicted by non-bolded signalling pathways described in references Shun *et al*, 2004; Yu *et al*, 2006; Jia *et al*, 2008a, 2008b; Wang *et al*, 2008) with involvement of prolonged activation of JNK. Our data show that JNK is activated by endoplasmic reticulum (ER) stress (Tiwary *et al*, 2010). Active JNK blocks IRS-1 signalling, thereby downregulating downstream survival signalling mediators, resulting in the activation of proapoptotic mediators Bad and caspase 9, leading to promoting mitochondria-dependent apoptosis. Combinations of suboptimal levels of *α*-TEA plus mTOR or MEK inhibitors act synergistically to induce apoptosis by reduction of AKT- and ERK-mediated inhibition of proapoptotic factors Bad and caspase 9.

To determine the effects of inhibition of members of the PI3K pathway on *α*-TEA-induced apoptosis, HCC-1954 and MCF-7 cells were cultured with 10 and 20 *μ*M *α*-TEA, respectively, plus 1 *μ*M PI3K inhibitor (PI3KI) wortmannin, 10 *μ*M MEK inhibitor (MEKI) U01260, and 50 nM mTOR inhibitor (mTORI) rapamycin for 18 h. Single treatments with *α*-TEA or inhibitors induced low levels of apoptosis, whereas combinations of *α*-TEA plus each of the three inhibitors individually significantly enhanced induction of apoptosis in both cell types in comparison with single treatments (Figure 5A and B). Both *α*-TEA and PI3K inhibitors reduced levels of pAKT (Ser-473), pERK1/2, and pmTOR (Ser-2448), and the combination of *α*-TEA+PI3K inhibitor acted cooperatively to enhance PARP cleavage and to reduce levels of pAKT (Ser-473), pERK1/2, and pmTOR (Ser-2448) when compared with individual treatments or control (Figure 5C). Both MEK and mTOR inhibitors enhanced *α*-TEA-induced PARP cleavage and cooperatively enhanced *α*-TEA inhibition of pERK1/2 and pmTOR, respectively (Figure 5D). Combinations of *α*-TEA+AKT1 or 2 siRNA acted cooperatively to induce apoptosis (Figure 6E) and inhibit pAKT (Ser-473) and total AKT1 and AKT2, respectively, as well as pmTOR (Ser-2448), but had no effect on basal levels of pERK1/2, showing that the ERK signalling pathway is independent of AKT signalling (Figure 5F). Taken together, data in Figure 5 show that *α*-TEA can downregulate the basal levels of active AKT, ERK1/2, and mTOR, and can act cooperatively with either chemical- or genetic-based inhibitors of these prosurvival mediators to enhance breast cancer cell death.

### *α*-TEA blocked the ability of MEK and mTOR inhibitors to induce increased levels of pAkt and IRS

It has been reported that MEK and mTOR inhibitors induce increased levels of phospho-Akt via negative feedback regulation of IRS (Wan *et al*, 2007; Jiang *et al*, 2009). As expected, MEK and mTOR inhibitors induced increased levels of pAKT and IRS-1, an outcome that limits their clinical anticancer efficacy; importantly, co-treatment with *α*-TEA was able to block this counterproductive increase in these potent prosurvival mediators (Figure 5G).

Based on these data, we hypothesise a signalling pathway depicting cross-talk between survival and death signalling pathways in *α*-TEA alone and *α*-TEA+MEK or mTOR inhibitor-induced apoptosis in human breast cancer cells (Figure 6). In the untreated cells, IGF/IGFR via downstream mediator IRS-1 regulates two distinct signalling pathways (PI3K and Ras) that contribute to basal levels of activated AKT, ERK, and mTOR. Activated AKT and ERK1/2 phosphorylate proapoptotic mediators Bad and caspase-9, thereby inhibiting their proapoptotic actions and thus enhancing cancer cell survival. Both ERK and mTOR trigger a negative feedback loop via their substrate p70S6K that downregulates IRS-1 signalling. Previous studies (non-bolded signalling pathway) have shown that *α*-TEA induces apoptosis via upregulation of cell surface death receptor-mediated caspase-8 and mitochondrial-dependent pathways (Shun *et al*, 2004; Yu *et al*, 2006; Jia *et al*, 2008b; Tiwary *et al*, 2010). Previous studies have also identified JNK as a key player in *α*-TEA-induced apoptosis (Shun *et al*, 2004; Yu *et al*, 2006; Jia *et al*, 2008a, 2008b; Wang *et al*, 2008). In addition to signalling apoptosis via p73/Noxa (Wang *et al*, 2008) and a Fas/JNK positive feedback loop (Jia *et al*, 2008a, 2008b), we report here that JNK suppresses PI3K prosurvival signalling pathways via phosphorylation (inactivation and degradation) of IRS-1. As IRS-1 has an apical role in activation of AKT, ERK, and mTOR survival signalling pathways via PI3K, elimination of IRS-1 deprives the cells of these signalling mediators as well as restores proapoptotic activity by Bad and caspase-9 via their dephosphorylation, leading to cell death by apoptosis. In summary, the results reported in this study help clarify why *α*-TEA is such a potent apoptotic agent in human breast cancer cell lines, and suggest potential utility for *α*-TEA in combination therapy with ERK and mTOR inhibitors.

## Discussion

These studies demonstrate, for the first time, that *α*-TEA suppresses AKT- and ERK-mediated antiapoptotic events, namely, inhibitory phosphorylation of two proapoptotic factors Bad and caspase-9. Additionally, *α*-TEA suppression of AKT leads to decreases in mTOR activity, as measured by reduction in downstream mediators p4E-BP1 and p70S6K. Functional knockdown assessments indicate that *α*-TEA-mediated activation of JNK has a critical role in these events via downregulation of IRS-1. The following sequence of events are proposed for *α*-TEA-mediated apoptosis: *α*-TEA inhibits PI3K via JNK-mediated phosphorylation of IRS-1 at ser-307, resulting in inactivation of AKT/mTOR and Ras/ERK, which act cooperatively in *α*-TEA-induced mitochondrial-dependent proapoptotic events via activation of Bad and caspase-9. Especially noteworthy is that these studies showed that the harmful outcome of drug inhibition of either ERK or mTOR, namely, activation of AKT, could be prevented by combination treatment with *α*-TEA. In summary, data show that *α*-TEA suppression of IRS-1 not only has an important role in *α*-TEA-induced apoptosis but can also suppress activation of AKT induced by ERK and mTOR inhibitors, suggesting that *α*-TEA might improve clinical outcomes of treatment with ERK or mTOR inhibitors.

The pleiotropic effects of PI3K/AKT signalling on inhibition of apoptosis have been reported to be a major mechanism for drug resistance (McCubrey *et al*, 2007; Gibbons *et al*, 2009; Falasca, 2010). Therefore, identification of agents that can both block survival and induce death signalling pathways should aid development of strategies to sensitise drug-resistant breast cancer cells. *α*-Tocopherol ether-linked acetic acid has been reported to suppress AKT in prostate and ovarian cancer cells and that suppression of AKT contributes to *α*-TEA-induced apoptosis (Yu *et al*, 2006; Jia *et al*, 2008a, 2008b; Shun *et al*, 2010). In prostate cancer cells, *α*-TEA suppression of AKT causes activation of Fox-1, a proapoptotic transcription factor capable of triggering apoptosis via upregulation of Bim (Jia *et al*, 2008a, 2008b). In ovarian cancer cells, *α*-TEA suppresses c-FLIP and survivin protein expression via AKT-mediated events (Shun *et al*, 2010). In this study we report that *α*-TEA suppression of AKT via targeting IRS-1/PI3K leads to activation of proapoptotic Bad and caspase-9 in human breast cancer cells.

Bad is a proapoptotic member of the Bcl-2 family, which belongs to the BH3-only protein family comprising Bad, Bik, Bmf, Hrk, Noxa, tBid, Bim, and Puma (Kim *et al*, 2006). Bcl-2 associated death (Bad) promoter, Bcl-2 antagonist of cell death) is a proapoptotic factor that promotes apoptosis via forming heterodimers with prosurvival proteins Bcl-2 and Bcl-xL, preventing them from binding with Bax (Yang *et al*, 1995). Phosphorylation of Bad at Ser-112 and Ser-136 disrupts its association with Bcl-2 or Bcl-xl, promoting cell survival (Datta *et al*, 2000; Hayakawa *et al*, 2000). Phosphorylation of Bad at ser-112 and ser-136 has been proposed to be mediated by ERK/p90RSK1 and PI3K/AKT pathways, respectively (Hayakawa *et al*, 2000). Thus, the phosphorylation status of Bad at these critical serine residues serves as a determinant of either cell death or survival (Downward, 1999). Data showing that *α*-TEA reduces phosphorylation of Bad at both ser-112 and ser-136 sites suggest that the ability of *α*-TEA to reduce AKT and ERK activities contributes to restoration of Bad's proapoptotic function. Furthermore, data showing that inhibition of PI3K with wortmannin suppressed phosphorylation of Bad at both ser-112 and ser-136 supports a role for PI3K in regulating both AKT and ERK/p90RSK1 in these cells. Previous data from our lab showed that *α*-TEA induces mitochondria-dependent apoptosis via activation of Bax, a critical step in triggering mitochondria-dependent apoptosis (Yu *et al*, 2006; Jia *et al*, 2008b; Yu *et al*, 2010). Two other BH3-only proapoptotic Bcl-2 members, caspase-8-mediated tBid (active form of Bid) and p73-mediated Noxa, have been shown to be upregulated by *α*-TEA (Yu *et al*, 2006; Jia *et al*, 2008b; Wang *et al*, 2008; Yu *et al*, 2010). In this study, for the first time, we report that *α*-TEA induces Bad activation via inhibiting AKT and ERK. These data demonstrate that *α*-TEA triggers mitochondria-dependent apoptosis via targeting different Bcl-2-associated death promoters, namely, Bid, Noxa, and Bad, in cancer cell types.

Caspase-9, a mitochondria-mediated initiation caspase, is directly activated by Apaf-1 and cytochrome *c* and triggers activation of execution caspases 3, 6, and 7, leading to DNA fragmentation and cell death (Li *et al*, 1997). It has been reported that caspase-9 activity is regulated by phosphorylation (Cardone *et al*, 1998). AKT phosphorylates caspase-9 at Ser-196, leading to inactivation of caspase-9 (Cardone *et al*, 1998). Therefore, caspase-9 is another target for AKT to prevent cells from undergoing apoptosis. Thus, *α*-TEA suppression of AKT phosphorylation of caspase-9 at ser-196 contributes to *α*-TEA-induced mitochondria-dependent apoptosis.

Mammalian target of rapamycin is a downstream mediator of PI3K/AKT signalling, regulating proliferation, survival, mobility, and angiogenesis via targeting p70S6 kinase (p70S6K) and 4E-BP1 in breast cancers that exhibit constitutively activated PI3K/AKT signalling (Bjornsti and Houghton, 2004). Accumulating evidence suggests that PI3K/AKT/mTOR promote breast cancer cell survival and resistance to chemotherapeutics such as trastuzumab (a blocking antibody to Her-2) and tamoxifen (Hynes and Dey, 2009; Ghayad *et al*, 2010). The mTOR inhibitors rapamycin and rapamycin analogues (CCI-779, RAD001, and AP23573) have exhibited impressive growth inhibitory effects against a broad range of human cancers, including breast cancer, in preclinical and early clinical studies (Chan, 2004; Vignot *et al*, 2005). In this study, we demonstrate that *α*-TEA functions as an mTOR inhibitor, capable of suppressing mTOR by decreasing constitutively activated mTOR (phosphorylated status of mTOR) and its downstream mediators p70S6K and 4E-BP1. In addition, our data show that *α*-TEA not only enhances rapamycin suppression of mTOR and induction of apoptosis, but also suppresses rapamycin-mediated feedback activation of AKT, providing a rationale for developing a combination regimen of mTOR+*α*-TEA for breast cancer treatment.

Insulin receptor substrate-1 is an adaptor protein important for the insulin receptor and IGF-1 receptor signal transduction to downstream targets, including PI3K (Surmacz, 2000; Valentinis and Baserga, 2001). It has important roles in maintaining insulin sensitivity in adipocytes and cell growth in cancer cells (Hartley and Cooper, 2002). Its activity is positively and negatively regulated via its phosphorylation at different sites by not only ligand-activated cell surface receptors but also by different intracellular Ser/Thr protein kinases, including mTOR, ERK, protein kinase C, and AMP-activated protein kinase, as well as JNK (De Fea and Roth, 1997; Ozes *et al*, 2001; Rui *et al*, 2001; Horike *et al*, 2003; Hiratani *et al*, 2005; Mingo-Sion *et al*, 2005). Insulin receptor substrate-1 Ser-307 lies near the phospho-tyrosine binding domain in IRS-1 and confers an inhibitory effect on both insulin and IGF-1 signalling (Greene *et al*, 2003). Activation of JNK has been established as a stress-mediated inducer of insulin resistance in diabetic animal models via phosphorylation of IRS-1 at Ser-307, leading to inactivation of IRS-1 by interfering with the interaction of the insulin receptor and IRS-1 and promoting IRS-1 degradation (Mamay *et al*, 2003). An inhibitory effect of JNK on IRS-1 activity via phosphorylation at ser-307 in human breast cancer cells has also been reported in Taxol treatments (Mamay *et al*, 2003). In this study we report that *α*-TEA functions as an IRS-1 suppressor in human breast cancer cells via JNK-dependent phosphorylation of IRS-1 at ser-307. Thus, *α*-TEA-mediated phosphorylation of IRS-1 at ser-307 is correlated with downregulation of total protein levels of IRS-1, and both *α*-TEA-mediated phosphorylation of IRS-1 and downregulation of total protein level of IRS-1 are JNK dependent, suggesting that *α*-TEA downregulation of total protein level of IRS-1 may be subsequent to phosphorylation of IRS-1 at ser-307, as phosphorylation of IRS-1 at ser-307 has been reported to decrease IRS-1 stability (Greene *et al*, 2003). Mammalian target of rapamycin and ERK have been reported to negatively regulate IRS-1 via their downstream mediator p70S6K (Wan *et al*, 2007; Jiang *et al*, 2009), providing a negative feedback mechanism for turning off activation of AKT (Sun *et al*, 2005). Thus, inhibitors of MEK/ERK and mTOR produce a counterproductive increase in pAKT via loss of the negative feedback control of IRS-1 levels, which limits their clinic applications. It is highly significant that *α*-TEA functions as an inhibitor of not only ERK and mTOR, but also of AKT. It is also highly significant that *α*-TEA in combination with ERK and mTOR inhibitors blocks inhibitor-induced increases in pAKT. The potent anticancer actions by *α*-TEA appear to be because of its ability to inhibit IRS-1/PI3K via activation of JNK.

c-Jun N-terminal kinase, a proapoptotic mediator, has been reported to be activated by *α*-TEA and involved in *α*-TEA-induced Fas and Fas L protein expression in prostate cancer (Jia *et al*, 2008b), as well as p73/Noxa protein expression (Wang *et al*, 2008) and CHOP/DR5 protein expression (Tiwary *et al*, 2010) in breast cancer cells. In this study we report that JNK suppressed pro-survival PI3K and its downstream mediators via targeting IRS-1. These data demonstrate that JNK has a critical pleiotropic role in *α*-TEA-induced apoptosis via both proapoptotic and antisurvival mechanisms. Mechanisms involved in the initiation of JNK activation in response to *α*-TEA are not fully understood. However, our recent data show that JNK is at least partially activated by endoplasmic reticulum (ER) stress in *α*-TEA-induced apoptosis (Tiwary *et al*, 2010). Following ER stress, Ire1 is released from GRP78 in ER, leading to activation of ASK1 via TRAF2 (Xu *et al*, 2005). Apoptosis signal-regulating kinase 1 (ASK1) has been shown to be critical in ER stress-mediated apoptosis and activation of JNK (Nishitoh *et al*, 2002). Thus, ER stress-mediated ASK1 is a possible upstream mediator for JNK activation.

In summary, the ability of *α*-TEA to target PI3K-mediated prosurvival factors via JNK-mediated inhibition of IRS-1 not only has a role in enhancing *α*-TEA-induced apoptosis by inhibition of AKT, ERK, mTOR, and their downstream mediators, but also suggests a novel means for preventing procancer impact of AKT activation induced by ERK and mTOR inhibitors.
